# Effects of Lumbar Spine Abnormality and Serve Types on Lumbar Kinematics in Elite Adolescent Tennis Players

**DOI:** 10.1186/s40798-020-00295-2

**Published:** 2021-01-11

**Authors:** Molly Connolly, Kane Middleton, Graeme Spence, Olivia Cant, Machar Reid

**Affiliations:** 1 Institute for Health and Sport, Victoria University, Melbourne, Australia; 2 Game Insight Group, Tennis Australia, Melbourne, Australia; 3 Sport and Exercise Science, School of Allied Health, Human Services and Sport, La Trobe University, Melbourne, Australia

**Keywords:** Biomechanics, Serve, Tennis, Lumbar spine

## Abstract

**Background:**

Low back pain (LBP) is pervasive among elite junior tennis players. Previous research has explored the relationship between serving mechanics and LBP, though the participants in these studies had already experienced LBP. Therefore, it is unclear whether their serving mechanics caused the LBP or are a result of having LBP. Thus, the purpose of this study was to compare the flat and kick serve kinematics of asymptomatic elite adolescent male and female tennis players with and without lumbar spine abnormalities. Twenty-four players (nine of which had confirmed lumbar spine abnormalities) carried out a series of flat and kick serves, while marker trajectories were recorded by a 3D motion capture system. Pelvis and lumbar spine kinematics (anterior/posterior tilt, lateral tilt, axial rotation and flexion/extension, lateral flexion and axial rotation respectively) were compared between players with and without lumbar spine abnormalities, genders, and serve types using a mixed-effects model. Exploratory data pertaining to the order and timing of key serve events was also collected.

**Results:**

Males had significantly greater posterior pelvis tilt than females during the drive phase of both flat (M, − 7.1 ± 5°; F, 4 ± 5.5°) and kick serves (M, − 8.6 ± 5.1°; F, 2.1 ± 5.8°). Independent of serve type, males also impacted the ball ~ 15 cm further into the court than females, while all players contacted flat serves significantly further forward (~ 17 cm). There were no effects for abnormality in the magnitude of pelvis and trunk kinematics. The order and timing of key serve events, however, did tend to differ between those with and without lumbar spine abnormalities. Players with abnormalities entered peak front knee flexion and initiated pelvis rotation earlier than players without abnormalities. Lastly, the timing of pelvis rotation was highly variable among females though not males.

**Conclusion:**

Pelvis and ball toss kinematics vary with gender and serve type but not necessarily abnormality in the elite adolescent serve. There is evidence to suggest that the order and timing of key serve events might help to identify those at risk of lumbar spine abnormalities; however, further research is needed to investigate the statistical significance of the timing of these events.

## Key Points

- Similar lumbar spine kinematics characterize the serves of elite junior tennis players with and without lumbar spine abnormalities. The utility of these measures for practitioners screening for at-risk serve technique would therefore seem limited.
- The evidence pointing to the timing of specific kinematic actions of serves being of interest to the presentation of lumbar spine abnormalities is instructive. Specifically, players with lumbar abnormalities entered peak front knee flexion and initiated pelvis rotation earlier. The sequencing of these types of actions can be observed by coaches and might introduce more balance into their analysis of both the magnitude and timing of serving kinematics.
- Select pelvis kinematics varied with gender during the serve. Male players were characterized by greater posterior pelvic tilt during the drive phase, yet this was not linked to the presentation of back pain. This finding adds to the body of literature that highlights the various ways in which different cohorts of players organize their serving mechanics.

## Introduction

Low back pain (LBP) is highly prevalent in tennis, particularly at the youth level [1]. Gescheit et al. [2] reported that the lumbar spine had the highest injury incidence among elite junior tennis players. It is also a pervasive problem in professional tennis, with Grand Slam tournament data revealing that lumbar pain is among the most common complaints of touring professionals [3]. While the incidence and severity of back pain has been reported a higher among male professionals (males 4.7 vs females 3.9 injuries per 1000 exposure hours), the reason for this gender-based difference remains unclear. More broadly though, magnetic resonance imaging (MRI) has shown that as many as 95% of asymptomatic players have radiological abnormalities at the lumbar spine, generally at the L4/L5 and L5/S1 levels [4, 5]. Clearly, lumbar spine pathologies are ubiquitous in tennis and actionable insight to limit their occurrence has largely eluded the sport.

A possible cause for low back pain in tennis players is the mechanics of the serve. The serve is the most important stroke in the game [6, 7], and its repeated high-speed three-dimensional rotation of the spine [7–10] has been widely implicated in lumbar injury. In particular, the kick serve has been shown to produce the highest forces on the back [11]. It is introduced to players as young as 13 years of age [11, 12], albeit more commonly among male adolescents. Historically, males have had higher incidences of lumbar injuries compared to females [2, 13]. That males learn the kick serve earlier and therefore experience those extreme loading conditions sooner might explain their comparatively higher incidence of lumbar injury. To the knowledge of the authors, the work of Campbell et al. [14] are the only studies to examine the influence of serve type (flat serve vs kick serve) on lumbar kinetics in elite adolescent males with and without low back pain. As this research was cross-sectional in nature and compared the serve mechanics of healthy players to players whom had previously suffered LBP, it is unclear whether the observed differences in the serve action were adaptive or maladaptive to pain. Thus, in order to gain a better understanding of the relationship between serving mechanics and LBP in tennis players, further research is required using asymptomatic tennis players with no history of LBP. Without better understanding the causes for low back pain in tennis, this pathology, if not managed carefully, could result in permanent structural damage. This in turn could lead to players withdrawing from the sport.

Research has not considered the female serve in the context of lumbar spine injury. This seems an unusual omission given female players have been reported to sustain fewer lumbar spine abnormalities/injuries. Indeed, previous work has found differences in the serving kinematics of junior elite male and female players [15]. For example, Connolly et al. [15] reported that male adolescents impacted the ball between 12 and 17 cm more laterally compared to females. To achieve these greater lateral impact positions, it is possible that the male players recruited more lateral flexion, which has been identified as a risk factor for LBP in tennis serving [14]. Verification of this more pronounced lateral flexion as well as other kinematic differences that might be implicated in the disparate presentation of LBP between male and female players remains a gap in the current sport medicine literature.

Given the prevalence and impact of LBP in junior tennis players, particularly males, the current study aimed to compare the effect of serve type, gender, and the presence of lumbar spine pars abnormalities on the kinematics and temporal sequencing of the serve in adolescent players. Our first hypothesis was that players with abnormalities would exhibit less dominant (right) side lumbar spine and pelvis rotation during the drive phase but greater non-dominant (left) side lateral flexion, lumbar spine rotation, pelvis rotation, and anterior pelvic tilt during the forward-swing phase than players without abnormalities. Our second hypothesis states that the male serve was characterized by increased lateral impact positions and drive phase lumbar extension as well as lumbar lateral flexion and posterior pelvic tilt while the female serve was anticipated to feature larger ball toss zeniths, ball toss drop distances, and peak knee flexion. Our third hypothesis was that the kick serve would see greater lumbar lateral flexion and extension compared to the flat serve as well as a smaller ball toss. Our final hypothesis expected the order and timing of pelvis, trunk, and ball toss kinematics to differ between the serves of those with and without abnormalities.

## Methods

### Participants

Twenty-four right-handed elite adolescent tennis players were recruited from the Tennis Australia National Academy. Participants included 14 males (age 13.6 ± 1.7 years, height 169.8 ± 12.7 cm, and weight 56.8 ± 13.1 kg) and 10 females (age 12.3 ± 1.3 years, height 160.5 ± 7.9 cm, and weight 51.6 ± 8.1 kg). Participants were excluded if they had either of the following: the participant had a previous bout of severe LBP (seven or more days missed training and/or competition due to LBP, similar to Ranson et al. [16]) with an accompanying MRI diagnosing a lumbar injury, the participant was ill, the participant had a performance inhibiting injury, or the participant experienced low back pain during testing. All players had recently undergone an MRI scan as part of an academy screening protocol which focused on the lumbar spine (L1/L2 to L5/S1). Based on their MRI screening results, participants were assigned to a group of those with pars abnormalities (in this study we included those with either a stress fracture and/or bone marrow edema (bone stress) at the pars interarticularis; designated by “P” from here on) or those without these abnormalities (designated by “NP” from here on). Participants in the P group were 13.2 ± 1.6 years old, 171.5 ± 12.3 cm tall, and had an overall body mass of 60.3 ± 11.2kgs, and the NP group was 13.0 ± 1.7 years old, 162.6 ± 10.3 cm tall, and had an overall body mass of 51.3 ± 10.4kgs. Ethics approval was obtained from the Victoria University Human Research Ethics Committee, and the participants and their parents provided voluntary written informed assent and consent respectively prior to their involvement in the study. This study was performed in accordance with the standards of ethics outlined in the Declaration of Helsinki.

### Procedure

**Fig. 1:**
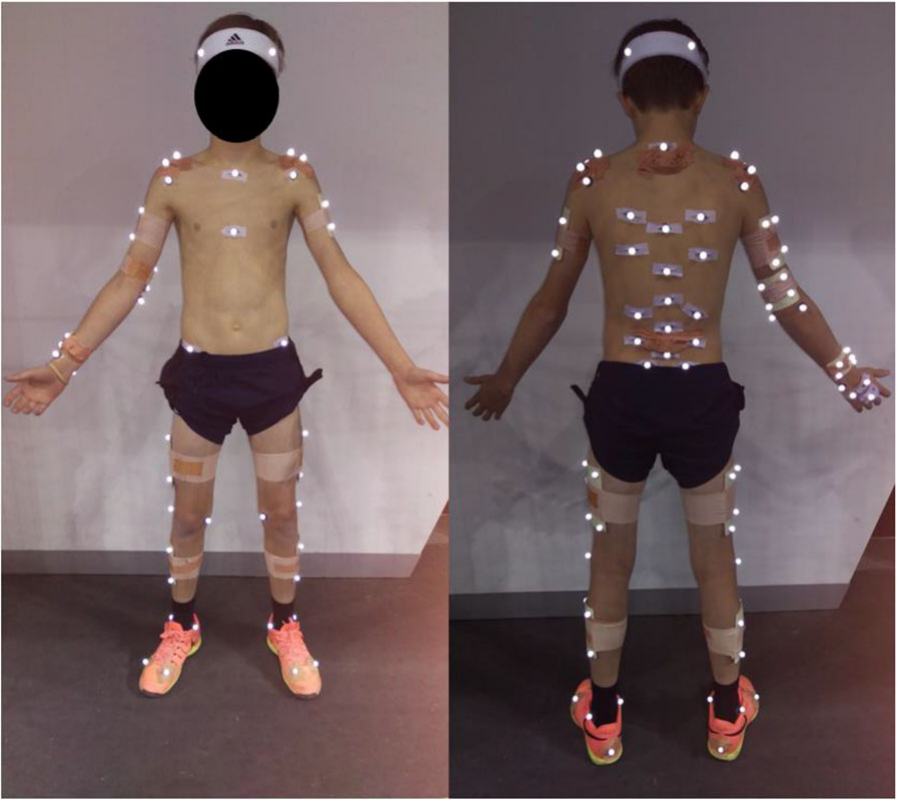
Retro-reflective markers on the body

A dynamic capture space (approximately 2 m (width) × 2 m (length) × 3 m (height)) was calibrated at the baseline using a 12-camera Vantage opto-reflective motion capture system (Vicon Motion Systems Ltd, Oxford, UK; 250 Hz). A global reference frame was set at the center mark on the baseline with positive X pointing toward the net, positive Y pointing directly leftward (along the baseline) when facing the net, and positive Z pointing directly upward. Prior to testing, participant height and mass were recorded followed by attaching retro-reflective markers. Retroreflective markers (12.7 mm diameter) and rigid plates with markers attached were then affixed to the participant’s skin (over specific positions or anatomical landmarks on the lower body, trunk and upper body) using double-sided tape and rigid sport tape (Fig. 1). Six markers were attached to the racquet (one at the butt, one at the tip, one either side of the widest part of the racquet and two at the throat of the racquet), and three round pieces of reflective tape were placed on the ball. Once the markers were attached, participants completed a self-directed warmup followed by a series of subject-specific calibration trials. Participants completed a series of serves aiming for a 1 m × 2 m target area bordering the “T” on the deuce court. Participants performed “Flat” serves (FS) until three serves landed in the target area and then followed with “Kick” serves (KS) at maximal intensity. Successful serves were defined as those that landed in the target area. Serving continued until three successful FS and KS were completed, adhering to prior established methods [14, 17].

### Data Preparation and Modeling

The data was processed and gaps in marker trajectories filled using VICON Nexus Software (Vicon Motion Systems Ltd, Oxford, UK). Trajectories were treated using a fourth order low-pass Butterworth filter at 15 Hz following a residual analysis and visual inspection of the data. Filtered anatomical, racquet, and ball data were modeled using a customized direct kinematic model [18–20]. The segment parameters for the upper body, thorax, and lumbar spine were defined based on previous research [21–23].

Joint angles (ankle, knee, hip, pelvis, and thorax) were expressed using the standard Euler Z-X-Y convention as per previous research [18, 24]. The pelvis and thorax were calculated relative to the global reference frame whereas the ankle, knee, and hip were relative to their anatomical reference frames. Lastly, the lumbar spine was calculated using the Euler Z-X-Y convention as reported by previous research [14, 18]. The sequence of rotations of the child segment relative to the parent segments were; flexion/extension, abduction/adduction, and internal/external rotation.

The dependent variables of interest included selected peak lumbar spine (flexion/extension, lateral flexion, axial rotation), pelvis (anterior/posterior tilt, pelvis obliquity and axial rotation), racquet (velocity and impact positions), and ball toss kinematics relevant to tennis serve performance and/or injury in past research [14, 17]. The timing of the peak lumbar spine kinematics were calculated relative to ball toss and ball impact and compared with other serving kinematics (these included beginning of pelvis rotation (anti-clockwise), racquet high point (RHP), racquet low point (RLP), ball zenith, peak right knee flexion, peak right knee extension, and when the toes leave the ground). Selected ball toss kinematics (peak ball toss height, three-dimensional impact position) were also measured relative to players’ height and will be described accordingly in the text. Kinematics were reported in the drive and forward-swing phase of the serve [25], and the temporal sequencing was described relative to ball toss and ball impact.

### Statistical Analysis

For each variable of interest, the mean kinematics of the three successful serves (per serve type) were used for analysis (Table 1). A mixed effects model identified the main effects for serve kinematic differences between the three comparison groups (P vs NP, male vs female, FS vs KS). As multiple comparisons were conducted, the alpha value was adjusted a priori to 0.01 to reduce the risk of type 1 error [26–28]. The temporal data in this study was exploratory and therefore no analysis was conducted on the timing of the serve events.

**Table 1:** Mean and standard deviation values for peak lumbar and pelvis kinematics for those with and without pars abnormalities

	Pars				No pars			
	Flat		Kick		Flat		Kick	
	Female	Male	Female	Male	Female	Male	Female	Male
**Drive phase**								
Lumbar extension (°)	− 4.9 ± 4.5	− 14.4 ± 9.0	− 5.4 ± 3.2	− 14.7 ± 9.6	− 9.7 ± 8.7	− 7.9 ± 8.4	− 9.6 ± 8.2	− 7.8 ± 9.3
Lumbar right lateral flexion (°)	− 6.1 ± 3.3	− 3.9 ± 5.3	− 6.3 ± 2.6	− 3.9 ± 4.7	− 1.8 ± 4.3	0.9 ± 3.8	− 2.6 ± 4.2	1.5 ± 4.1
Lumbar right axial rotation (°)	0.5 ± 2.8	− 1.1 ± 2.0	0.7 ± 3.1	− 1.3 ± 2.1	0.6 ± 2.1	− 0.2 ± 1.8	0.9 ± 2.2	− 0.4 ± 2.0
Trunk extension (°)	− 43.9 ± 1.0	− 20.5 ± 6.2	− 41.9 ± 1.5	− 21.1 ± 6.1	− 22.4 ± 13.7	− 25.0 ± 7.0	− 22.9 ± 15.8	− 26.9 ± 8.4
Trunk right lateral flexion (°)	20.4 ± 9.1	25.1 ± 11.0	21.2 ± 8.5	24.7 ± 12.0	29.2 ± 7.5	26.2 ± 9.7	29 ± 8.1	26.8 ± 10.3
Trunk right axial rotation (°)	− 31.6 ± 5.1	− 25.5 ± 15.9	− 28.5 ± 3.2	− 24.1 ± 14.9	− 23.3 ± 9.0	− 25.1 ± 10.7	− 23.8 ± 10.2	− 22.4 ± 11.4
Pelvis right rotation (°)	− 74.7 ± 4.1	− 94.9 ± 26.3	− 75.6 ± 7.8	− 105.3 ± 24.4	− 96 ± 13.7	− 102.5 ± 12.1	− 97.4 ± 15.2	− 109.9 ± 14.5
Pelvis posterior tilt (°)^^^	7.7 ± 1.5	− 8.6 ± 4.7	4.6 ± 3.7	− 10.3 ± 5.1	3.1 ± 5.7	− 5.5 ± 5.0	1.5 ± 6.1	− 7 ± 4.6
Pelvis obliquity (right down) (°)	3.3 ± 2.8	8.5 ± 4.0	3.6 ± 1.1	9.3 ± 4.1	7.1 ± 3.5	9.5 ± 7.0	7.1 ± 2.6	10.4 ± 8.2
Lumbar extension angular velocity (°/s)	− 189.5 ± 69.0	− 179.8 ± 112.8	− 156.8 ± 31.6	− 190.9 ± 134.2	− 253.3 ± 106.2	− 150.1 ± 42	− 237.4 ± 117.2	− 186.3 ± 76.6
Lumbar right lateral flexion angular velocity (°/s)	183.9 ± 22.1	200.5 ± 137.9	177 ± 27.8	203.5 ± 203.0	224.5 ± 60.1	146.8 ± 42.1	210.3 ± 61.7	165 ± 61.6
Lumbar right axial rotation angular velocity (°/s)	58.4 ± 36.5	54.4 ± 29.1	50.2 ± 26.9	54.8 ± 33.8	62.8 ± 31.9	59.3 ± 27.1	63.8 ± 35.5	53.1 ± 19.9
**Forward**-**swing phase**								
Lumbar flexion (°)*	10.4 ± 1.4	2.6 ± 3.7	11 ± 1.7	3.3 ± 3.3	5.8 ± 4.6	6.3 ± 4.4	7.6 ± 4.7	6.6 ± 4.4
Lumbar left lateral flexion (°)	− 19 ± 1.5	− 16.1 ± 5.2	− 18.8 ± 2.4	− 16.8 ± 5.2	− 16.9 ± 6.2	− 11.4 ± 5.2	− 17.3 ± 5.5	− 10.7 ± 5.2
Lumbar left axial rotation (°)	− 1.6 ± 2.4	− 1.5 ± 2.3	− 2.1 ± 1.7	− 1.7 ± 2.4	− 0.7 ± 2.3	− 1 ± 1.7	− 1 ± 2.4	− 1.3 ± 2.1
Trunk flexion (°)	5 ± 10.3	16 ± 8.0	5.7 ± 5.2	15.6 ± 12.1	7 ± 19.5	6.6 ± 12.9	13.5 ± 17.5	5.9 ± 11.3
Trunk left lateral flexion (°)	− 37 ± 10.6	− 38.5 ± 8.0	− 36.5 ± 9.6	− 40.1 ± 8.6	− 36.1 ± 8.9	− 35.4 ± 11.7	− 34.6 ± 10.7	− 37 ± 8.8
Trunk left axial rotation (°)	− 9 ± 3.2	− 9.5 ± 8.4	− 7.7 ± 5.7	− 11.6 ± 7.6	− 6.4 ± 13.4	− 6.1 ± 10.4	− 10.2 ± 12.2	− 6.5 ± 8.5
Pelvis left rotation (°)	− 4.2 ± 12.5	− 9.2 ± 14.5	− 8.8 ± 14.7	− 24.5 ± 16.5	− 2.3 ± 10.2	− 4.8 ± 12.8	− 13.5 ± 10.8	− 28.7 ± 20.7
Pelvis anterior tilt (°)*	31.4 ± 6.1	22.7 ± 7.9	28.7 ± 8.3	18.4 ± 8.4	33.7 ± 3.6	28.5 ± 9.9	27.7 ± 6.9	22.6 ± 10.5
Pelvis obliquity (left down) (°)*	− 27 ± 4.1	− 31.8 ± 6.7	− 30.6 ± 1.6	− 33.2 ± 7.1	− 21.6 ± 9.4	− 27.9 ± 3.4	− 25.9 ± 6.5	− 32 ± 5.0
Lumbar flexion angular velocity (°/s)	258.9 ± 44.8	289.5 ± 132.6	268.6 ± 57.7	316.4 ± 152.4	463 ± 360.1	241.3 ± 103.2	335.3 ± 72.8	227.3 ± 75.1
Lumbar left lateral flexion angular velocity (°/s)	− 177.4 ± 78.6	− 124.1 ± 91.0	− 218.5 ± 71.6	− 162.7 ± 130.1	− 198.8 ± 88.8	− 152.1 ± 44.2	− 204 ± 113.9	− 134.4 ± 32.9
Lumbar left axial rotation angular velocity (°/s)	− 84.3 ± 26.7	− 93.9 ± 56.0	− 90.5 ± 40.4	− 84.7 ± 56.3	− 125.9 ± 129.4	− 79.9 ± 75.0	− 83.7 ± 33.1	− 73.9 ± 41.8

*Significant main effect for serve type (*p*< 0.01)

^^^Significant main effect for sex (*p*< 0.01)

## Results

### The Effect of Lumbar Abnormalities on Serve Kinematics

The pelvis and trunk kinematics that characterized the serves of the P and NP groups were comparable (Table 1). Lumbar right lateral flexion was the most disparate between the two groups—with higher flexion in the NP group (*p* = 0.03). Ball toss kinematics and racquet-head velocity were also comparable between P and NP groups.

### The Effect of Gender on Serve Kinematics

Posterior pelvic tilt during the drive phase was significantly greater in males than females in both the flat (~ 11° difference) and kick serves (~ 10° difference, *p*< 0.01, Table 1). Peak right (back) and peak left (front) knee and hip extension angular velocities were comparable.

Serve impact position was further forward in both the flat (male 57 cm, female 42 cm) and kick (male 40 cm, female 25 cm) male serves (*p*< 0.01, Table 2). Differences in the vertical displacement of the ball toss were also observed with peak relative ball toss height significantly higher in the female serve (*p*< 0.01), leading to significantly larger ball drop distances (~ 27 cm for flat and kick).

**Table 2:** Mean and standard deviation values for peak lower limb and ball toss kinematics

	Pars				No pars			
	Flat		Kick		Flat		Kick	
	Female	Male	Female	Male	Female	Male	Female	Male
Front knee angle (°)	77.3 ± 8.2	68.2 ± 5.7	78.9 ± 9.5	67.7 ± 5.7	66.6 ± 9.6	67.9 ± 8.0	65.9 ± 8.9	69.2 ± 8.8
Back knee angle (°)	73.0 ± 9.6	79.9 ± 10.2	76.2 ± 11.8	79.3 ± 10.8	77.8 ± 8.5	73.1 ± 11.7	75.4 ± 11.2	73.6 ± 14.3
Front hip extension angular velocity (°/s)*	− 186 ± 12.7	− 193.9 ± 76.1	− 198.9 ± 19.4	− 199.2 ± 69.6	− 255.2 ± 54.6	− 214.1 ± 72.3	− 236.3 ± 53.8	− 203.7 ± 66.4
Front knee extension angular velocity (°/s)*	− 452.5 ± 28.8	− 462.4 ± 161.3	− 505.8 ± 37.3	− 507.2 ± 158.2	− 581.9 ± 129.1	− 478.7 ± 98	− 633.5 ± 147.4	− 520.7 ± 121.7
Back hip extension angular velocity (°/s)*	− 175 ± 34.5	− 230.2 ± 84.8	− 173.5 ± 8.9	− 236.3 ± 90.1	− 291.3 ± 68.6	− 240.1 ± 87.9	− 262.7 ± 86	− 231.6 ± 85
Back knee extension angular velocity (°/s)	− 510.1 ± 41.1	− 599.6 ± 149.7	− 510.8 ± 32.8	− 596.8 ± 123.9	− 615.7 ± 107.4	− 557.4 ± 90.4	− 634.1 ± 136	− 568.2 ± 94.9
LHJCL velocity (m/s)*	1.4 ± 0.1	1.4 ± 0.4	1.4 ± 0.1	1.5 ± 0.4	1.6 ± 0.2	1.4 ± 0.2	1.6 ± 0.3	1.5 ± 0.3
RHJCL velocity (m/s)	1.6 ± 0.1	2 ± 0.4	1.8 ± 0.1	2.2 ± 0.4	2 ± 0.2	2 ± 0.2	2.1 ± 0.4	2.2 ± 0.3
Racquet velocity X (m/s)*	32.1 ± 2	37.3 ± 5.3	30.4 ± 1.2	32.8 ± 4.2	34.7 ± 3.2	37.2 ± 5.9	30.3 ± 3.5	31.5 ± 4.9
Racquet velocity Y (m/s)	6.5 ± 1.5	8.3 ± 1.8	8.8 ± 2.1	8.4 ± 2.5	5.6 ± 2.3	6.9 ± 1.9	5.9 ± 2.3	7.7 ± 2
Racquet velocity Z (m/s)*	23.3 ± 1.6	24.7 ± 2.1	22.8 ± 0.9	24.2 ± 2.9	24 ± 3.1	24.3 ± 3.8	22.8 ± 3.3	23 ± 3.7
Toss height (cm)	319 ± 16.0	317.1 ± 33.5	319.1 ± 8.0	315.9 ± 23.0	331.6 ± 20.7	306.5 ± 26.1	332.8 ± 25.2	305.1 ± 25.2
Relative toss height (ratio)^^^	0.51 ± 0.01	0.55 ± 0.06	0.51 ± 0.01	0.55 ± 0.05	0.48 ± 0.04	0.54 ± 0.06	0.48 ± 0.05	0.55 ± 0.06
Lateral impact position (cm)*	− 24.3 ± 6.0	− 31.1 ± 18.8	− 38.8 ± 11.2	− 44.5 ± 24.1	− 4.5 ± 25.5	− 19.8 ± 21.1	− 25.1 ± 22.0	− 43.0 ± 24.1
Relative lateral impact position (ratio)*	− 0.15 ± 0.04	− 0.18 ± 0.1	− 0.24 ± 0.08	− 0.25 ± 0.13	− 0.03 ± 0.16	− 0.12 ± 0.12	− 0.16 ± 0.13	− 0.26 ± 0.14
Forward impact position (cm)*^^^	− 38.0 ± 13.6	− 53.2 ± 31.8	− 31.1 ± 11.1	− 42.4 ± 12.2	− 42.8 ± 10.8	− 61.6 ± 11.9	− 23.9 ± 13.7	− 38.1 ± 16.1
Relative forward impact position (ratio)*^^^	− 0.24 ± 0.09	− 0.3 ± 0.18	− 0.2 ± 0.08	− 0.24 ± 0.06	− 0.27 ± 0.07	− 0.37 ± 0.08	− 0.15 ± 0.09	− 0.23 ± 0.09
Impact height (cm)	243.8 ± 5.8	257.7 ± 18.2	242.0 ± 8.0	257.0 ± 19.5	246.0 ± 11.2	250.9 ± 15.8	246.9 ± 12.5	249.1 ± 16.2
Relative impact height (ratio)	0.66 ± 0.02	0.69 ± 0.06	0.67 ± 0.01	0.68 ± 0.02	0.65 ± 0.02	0.66 ± 0.02	0.65 ± 0.02	0.67 ± 0.03
Drop distance (cm)^^^	75.2 ± 10.9	59.4 ± 36.4	77.1 ± 3.7	58.9 ± 26.7	85.6 ± 20.8	55.6 ± 24.8	85.9 ± 26.3	56.0 ± 22.0

*LHJCL* left hip joint center linear, *RHJCL* right hip joint center linear

*Significant main effect for serve (*p*< 0.01)

^^^Significant main effect for sex (*p*< 0.01)

### The Effect of Serve Type on Serve Kinematics

Serve type had no effect on the lumbar spine kinematics during the drive phase but some differences emerged during the forward-swing phase of the serve. All participants flexed their lumbar spines more in the kick serve forward-swing. The flat serve, conversely, was characterized by greater anterior pelvic tilt and less pelvis obliquity (left down, ~ 3° difference, *p*< 0.01). The extension angular velocity profile of the lower limbs was interesting between serves, with higher magnitudes of front and back hip extension angular velocities (7°/s and 10°/s respectively) (*p*< 0.01) observed in the flat serve but the front knee extension more dynamic in the kick serve (*p*< 0.01, Table 2).

At impact, both in absolute and relative terms, the kick serve was impacted significantly further across the body and the kick serve was hit significantly further into the court. Peak forward and vertical racquet velocities were ~ 5 m/s and ~ 1 m/s faster in the flat serve respectively (*p*< 0.01).

### Temporal Kinematics

**Fig. 2:**
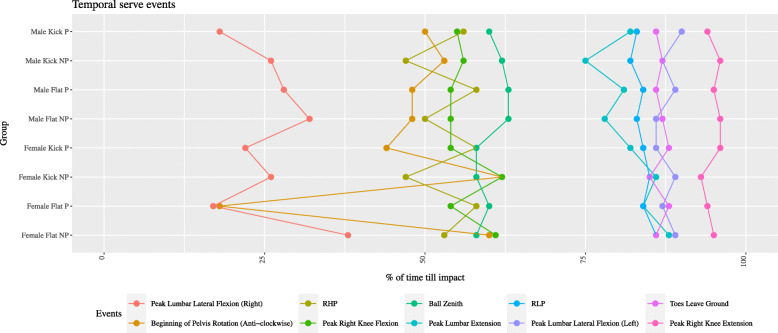
Chart displaying when key serve events occur throughout the serve as a percentage of time. RHP, racquet high point; RLP, racquet low point

When observing the exploratory data, a comparison between key serve events revealed differences between the P and NP groups. Peak front knee flexion and the commencement of pelvis left rotation (in almost all cases) preceded racquet high point (RHP) in the P serve (Fig. 2). Peak right lumbar lateral flexion also occurred earlier in the P group (Fig. 1a). This resulted in a substantially longer time lag between peak right lumbar lateral flexion and RHP in the P group (Fig. 2). The initiation of pelvis left rotation was highly variable in the female serve but stable among the male serve (Fig. 2).

## Discussion

This is the first study to investigate the relationship between lumbar spine abnormalities in asymptomatic elite adolescent players and serve kinematics. The aim of this study was to compare the effect of lumbar spine pars abnormalities, gender, and serve type on the kinematics and temporal sequencing of the serve in adolescent tennis players. No differences in peak lumbar spine, pelvis, or ball toss kinematics were observed between those with and without abnormalities. However, some differences in peak lumbar spine, pelvis, and ball toss kinematics existed between gender and between serve types. This study was also novel in its comparison of the effect of gender and serve type on the kinematics of the adolescent serve.

### The Relationship Between Lumbar Abnormalities and Serve Kinematics

Surprisingly, the lumbar spine kinematics were comparable in magnitude, independent of the presence of abnormalities. Consequently, our first hypothesis was rejected. These findings contrast with previous research that has inferred a link between serve kinematics and low back pain among adolescent male tennis players [14]. Despite being informed by previous research [14], our hypothesized reduction in lumbar and pelvis rotation in both the drive and forward-swing phases of those without abnormalities was not substantiated. Unexpectedly, lumbar left lateral flexion, lumbar and pelvis left rotation, and pelvic anterior tilt were also comparable in the forward-swing phase. While discrete kinematics are valuable in determining peak/moment-in-time differences, there are shortcomings of analyzing these values in isolation. For example, while there were no observed differences in peak lumbar kinematics, the order and timing of the kinematics did vary considerably between groups. These variations might prove instructive for coaches when identifying players at risk of lumbar abnormalities [29], whereby the temporal features of serving might be observed via high speed video or sensor-based technologies [30]. Specifically, players with lumbar spine abnormalities tended to enter peak front knee flexion and initiate anti-clockwise pelvis rotation before RHP, which is in contrast to players without spine abnormalities. These two kinematics could be detected and monitored by coaches using the abovementioned technologies. It is worth noting however that further research investigating the statistical significance of the relationship between the order and timing of key serve events with lumbar spine abnormalities is needed.

Lastly, the variation in age and skill level likely contributed to our findings, that is, the younger participants in our study displayed large amounts of variation in their kinematics, potentially indicative of still maturing technique.

### The Relationship Between Gender and Serve Kinematics

As expected, there were kinematic differences between the junior male and female serve. Peak posterior pelvic tilt was ~ 11° greater in male players during the drive phase of both the flat and kick serve. Most females adopted a more upright trunk posture during the ball toss (between ~ 3 and 4° more trunk extension), a probable by-product of these female players maintaining a neutral or anteriorly tilted pelvis during the drive phase compared to males. This trunk alignment tended to coincide with more pronounced peak front knee flexion, which saw female players assume a squat-like or more vertical (up-down) serve than male players.

Males made serve impact significantly further into the court on the flat (~ 16 cm) and kick (~ 15 cm) serves, even when held relative to their standing stature. The forward impact location of the adolescent male flat serve was similar to past research [17, 27] that has found junior and adult players to impact the ball ~ 52-58 cm forward of the front toe. The adolescent female players in the current study however tossed the ball up to 20 cm closer to the baseline than previous descriptions of the adolescent female serve [28]. It is possible that this was linked to the adoption of the abovementioned upright trunk position during the drive phase, which likely contributes to a reduced shoulder-over-shoulder rotation.

Interestingly, males impacted the flat serve 25 cm and kick serve 44 cm to the left of their front toe, which is substantially higher than some elite adult players [17]. If we assume that the average standing height of male player in past research is 183 cm, then the difference in relative lateral impact position (adults 0.19; adolescents 0.26) is even more extreme. Although speculative, we expect that this leftward positioning of the ball relates to a combination of the heightened need to impart spin to the ball to clear the net as well as introduction of the kick serve at this age. Importantly, for players to position themselves in this way, there is likely to be compensation elsewhere. For example, pelvis obliquity (where the right hip was vertically higher than the left) was much higher than reported in other elite junior populations [14]. This appeared to result in players’ bodies being rotated laterally, potentially explaining why players in this study impacted the ball further across their body compared to similar previously studied populations [14]. This type of alignment of the body might be injurious if unconstrained and is worth coaches and health professionals monitoring.

### The Relationship Between Serve Type and Serve Kinematics

Flat and kick serve kinematics were notably different, largely supporting our third hypothesis. The kick serve displayed increased lumbar flexion and pelvis obliquity (left down), suggesting that players adjust their sagittal plane lumbar kinematics and pelvis position to achieve laterally displaced impacts. Similar to the observed differences in impact position based on gender, serve type also significantly alters the relationship between ball and racquet at impact. As with previous research in the adult game [17], players in this study made flat serve impact significantly further forward (51 cm vs kick 34 cm) and with higher horizontal velocity.

Interestingly, in contrast to previous work in elite tennis players [31], peak vertical racquet velocity was significantly higher for the flat serve. Conversely, previous work has established that vertical racquet velocities are higher for second serves in order to impart topspin on the ball [31]. A combination of comparatively smaller player heights and inexperience, as these junior players were likely only recently introduced to the kick serve, present as the most likely explanations of this finding.

### Temporal Kinematics

The order and timing of key serve events was different between the P and NP groups, upholding our final hypothesis. Specifically, peak right lumbar lateral flexion and pelvis left rotation as well as peak front knee flexion occurred prior to RHP in players with abnormalities indicating possible early initiation of leg drive. Indeed, this difference in sequencing coupled with their earlier engagement of peak right knee flexion meant that the RHP of players with abnormalities was substantially different to those without abnormalities. The importance of RHP to the serve’s rhythm has been emphasized previously [27], and the lower (8 cm) ball zenith of the P group afforded them less time to self-organize in order to impact the ball. Keeping in mind that the players in the P group were asymptomatic, it is possible that the difference in their timing of serve events might lead to different loading of the spine which may ultimately lead to LBP. Therefore, more work is needed to explore the differences in serve sequences in players with lumbar abnormalities and whether these serve sequences result in additional spinal loading.

Female players with abnormalities tended to reach peak lumbar extension and peak lumbar left lateral flexion earlier than players without abnormalities. This is likely related to their reduced lumbar extension and commencement of pelvis rotation prior to RHP. As the lumbar spine is extended during the drive phase, increasing the duration of time spent in lumbar extension may be deleterious due to the amount of stress placed on the spine in this position [8].

Sample size was a limitation in this study due to the strict criteria and limited number of elite adolescent athletes available. This in turn resulted in participants’ age varying. This study also recruited players who reported as pain free at the commencement of the study.

## Conclusion

The magnitude of discrete pelvis and lumbar spine kinematics, during the drive and forward-swing phases of the flat and kick serve, did not discriminate between elite adolescent players with and without lumbar abnormalities. Various kinematic differences were however observed between the male and female adolescent serve, which is interesting given that low back injury is more prevalent in male players. Significantly, in a departure from previous work, this study investigated and observed differential timing in the lower limb, pelvis, and lumbar spine kinematics in the serves of players with and without lumbar abnormalities. This provides some initial evidence suggesting that the way in which players arrive into RHP in their serves may be a risk factor in low back pain. This information is useful as through the use of cameras, coaches could film sessions and observe the timing of key serve events to help identify serving patterns that might lead to LBP in junior tennis players. Lastly, this information informs both coaches and medical staff of movements in the serve that could be associated with the onset of LBP and thus will help in establishing prevention strategies.

## Data Availability

The dataset for this study will not be publicly available due to an IP agreement between Tennis Australia and Victoria University

## Abbreviations

LBP: Low back pain
MRI: Magnetic resonance imaging
FS: Flat serve
KS: Kick serve
RHP: Racquet high point
RLP: Racquet low point
P: Players with pars interarticularis abnormalities
NP: Players without pars interarticularis abnormalities
LHJCL: Left hip joint center linear
RHJCL: Right hip joint center linear
